# Draft Genome Sequence of a New Pseudomonas sp. Strain, ef1, Associated with the Psychrophilic Antarctic Ciliate Euplotes focardii

**DOI:** 10.1128/MRA.00867-19

**Published:** 2019-10-10

**Authors:** Kesava Priyan Ramasamy, Andrea Telatin, Matteo Mozzicafreddo, Cristina Miceli, Sandra Pucciarelli

**Affiliations:** a School of Biosciences and Veterinary Medicine, University of Camerino, Camerino, MC, Italy; b Quadram Institute Bioscience, Gut Microbes and Health Institute Strategic Program, Norwich Research Park, Norwich, United Kingdom; Loyola University Chicago

## Abstract

We announce here the draft genome sequence of a new *Pseudomonas* strain, named *Pseudomonas* sp. strain ef1, associated with the cold-adapted Antarctic ciliate Euplotes focardii. The genome sequence is 6,228,167 bp long with a G+C content of 59.7%.

## ANNOUNCEMENT

The genus *Pseudomonas* is ubiquitous in aquatic and terrestrial environments and metabolically diverse, including the capacity to remove toxic heavy metals (1) and to degrade various aromatic hydrocarbons (2, 3). The bacterial strain reported here was isolated from a consortium associated with the cold-adapted ciliate Euplotes focardii maintained in the laboratory at 4°C (4, 5). The present study aimed to isolate and characterize metal-resistant bacteria that can be useful for biosorption. For strain isolation, the logarithmically growing *E. focardii* cultures were harvested by centrifugation at 3,000 rpm for 10 min, and the pellet was suspended with sterile seawater. The suspension was sonicated for 5 to 10 seconds at a pulse rate of 6 V. The total cell extract (200 μl) was inoculated directly into lysogeny broth (LB) agar medium (1% tryptone, 0.5% yeast extract, 1% NaCl, and 1.5% agar) supplemented with the final concentration of filter-sterilized 2 mM copper (II) chloride (Sigma-Aldrich) solution and incubated at 4°C for 1 week. Morphologically different colonies were picked and routinely subcultured onto LB agar plates to obtain a pure culture. The isolated strain was stored as a stock in 25% glycerol at –80°C for further use. Prior to DNA extraction, a single colony was grown in LB broth, and genomic DNA was extracted using the commercial PureLink genomic DNA isolation kit (Invitrogen) according to the manufacturer’s instructions. Genomic DNA integrity was checked with gel electrophoresis and then quantified with the Qubit fluorometer (double-stranded DNA [dsDNA]) assay. A whole-genome shotgun library was prepared, starting from 1 ng of genomic DNA, using the Illumina Nextera XT kit. Whole-genome sequencing was performed by Illumina MiSeq 2 × 300-bp sequencing at BMR Genomics, Padua, Italy, and generated a total of 1,358,254 reads (12-fold coverage). All sequence reads were quality checked using FastQC 0.11.1 (6) and assembled using SPAdes 3.6 (7) with the following parameters: k-mer values of 21, 33, 55, 77, 99, and 127 and –careful. Default parameters were used for all software unless otherwise specified. Genome sequences were annotated with Prokaryotic Genome Annotation Pipeline (PGAP) version 4.8 (8). The assembly consisted of 72 contigs (*N*_50_, 242 kbp), 5,587 predicted coding DNA sequences (CDSs), 6 rRNA operons (5S, 16S, and 23S), 62 tRNAs, and 4 noncoding RNAs. Compared with the reference Pseudomonas koreensis D26 (GenBank accession number CP014947), 71% of the assembly bases were aligned (4.5 Mbp) with OrthoANI (9) with a value of 99.94%. This genome harbored a set of copper resistance and copper transporting genes which may be important for the survival of *Pseudomonas* sp. strain ef1 in the presence of copper. Genome analyses revealed the presence of aromatic hydrocarbon degradation genes, such as homogentisate 1,2-dioxygenase (locus tag FEE99_00455), protocatechuate 3,4-dioxygenase (locus tag FEE99_02300), and salicylate hydroxylase (locus tag FEE99_04700) (10). Based on the functional annotation in the Rapid Annotations using Subsystems Technology (RAST) 2.0 server (11), we predicted a total of 107 genes potentially involved in the metabolism of aromatic compounds. The draft genome sequence of *Pseudomonas* sp. strain ef1 provides new insights for a better understanding of ecologically important degradation genes. Furthermore, it provides the knowledge of enzymes and other protein sequences able to function at a constant temperature of 4°C.

### Data availability.

The GenBank/EMBL/DDBJ accession number for the 16S rRNA gene sequence of *Pseudomonas* sp. strain ef1 is MH177769. This whole-genome project for *Pseudomonas* sp. strain ef1 has been deposited in GenBank under the accession number VAUR00000000. Raw reads are available under the SRA accession number SRR9712345.
